# Global Earth Observations for Health

**DOI:** 10.1289/ehp.113-a738

**Published:** 2005-11

**Authors:** Charles W. Schmidt

Every day, Earth-observing satellites outfitted with remote-sensing technology generate vast data streams that scientists use to study the biosphere—the part of the Earth and its atmosphere that can support life. These orbiting systems are rapidly advancing studies of climate change, weather, and other global phenomena. Now experts are looking for ways to put them to work in the field of environmental health research.

Recently, the NIEHS and the U.S. Environmental Protection Agency (EPA) united health and Earth scientists in a workshop charged with two key objectives. The first was to determine if observations of air quality and climate from space could be used as public health tools for research, policy decisions, and environmental and health planning. The second was to engage the NIEHS extramural research community in dialogue with remote-sensing data producers and organizers including the National Oceanic and Atmospheric Administration, the National Aeronautics and Space Administration, and the EPA. Together these experts explored ways to use Earth observation data in studies of air pollution and health.

## The NIEHS and Earth Observations

The workshop, titled “Global Earth Observations: Application to Air Quality and Health,” was held at the NIEHS campus on 1–2 August 2005, and was attended by several dozen academic and government scientists. “Health researchers already use ground-based measurements of air pollution [to assess human exposures], and the workshop provided a mechanism for them to consider if addition of remote-sensed data would improve their exposure assessment and analysis of disease outcomes,” says Sally Tinkle, a program administrator with the Cellular, Organs, and Systems Pathobiology Branch of the NIEHS Division of Extramural Research and Training. Tinkle, together with NIEHS program analysts Mary Gant and Mike Humble and EPA representatives Gary Foley, Valerie Garcia, and Andy Bond, organized the event and provided NIEHS scientific support.

The NIEHS plays a growing role in the use of this technology, in part through its membership in the U.S. Group on Earth Observations (USGEO), a standing committee that reports to the National Science and Technology Council’s Committee on Environment and Natural Resources. The USGEO recently drafted a 10-year strategic plan for applying Earth observations to health and environmental research, which was released by the White House on 6 April 2005. Tinkle is the NIEHS’s USGEO representative, and Gant leads the USGEO’s User Interface Working Group.

At the August workshop, speakers covered issues ranging from the strength and adequacy of remote-sensing data to new directions in satellite research, coverage with land-based monitoring networks, and the challenges of using spatial data to address air quality and health outcomes. Participants also split into working groups to identify potential demonstration projects for remote sensing in three areas of health research: respiratory disease, cardiovascular disease, and developmental biology. Outcomes in all three areas have been linked to air pollution.

## An Emphasis on Feasibility

Despite an initial focus on user needs in the area of remote-sensing data architecture—the way data are organized, stored, and made available to users—the workshop dialogue shifted frequently to feasibility issues. While the health scientists present found the technology intriguing, they raised questions about its potential for human exposure assessment.

Resolution limits were of particular concern. Remote sensing’s spatial resolution, for instance, is rarely less than a square kilometer, although there is increasing evidence that air pollution levels vary at much finer scales of resolution (for instance, city blocks). Temporal resolution can also be problematic, especially for polar-orbiting satellites, whose positions remain fixed as the Earth rotates beneath them (this is less of a problem for geostationary satellites, which orbit in sync with a particular location and thus image that area all the time).

Discussions also addressed methods for averaging pollution concentrations measured from space. Remote sensors measure pollution in atmospheric columns that extend to the outer edge of the stratosphere. Humans, however, are exposed to pollutants close to the Earth’s surface.

Finally, participants discussed limits on remote particulate measurements, which don’t extend below the 10-micron level and cannot distinguish between chemical species on particle surfaces. “All these factors contribute to the uncertainty of linking remote-sensing data to human effects,” says workshop participant Raymond Hoff, a professor of physics at The University of Maryland, Baltimore County.

According to Tinkle, feasibility discussions exposed the need for demonstration studies that layer remote-sensing data over existing ground-based pollution data sets. “This would permit us to determine if the addition of remote-sensing data improves the correlation of air pollution with adverse health events—such as asthma exacerbation and myocardial arrhythmias, for instance—in the area of respiratory and cardiovascular disease,” she says.

## Working Group Conclusions

Peggy Reynolds, an investigator with the Environmental Health Investigations Branch of the California Department of Health Services, moderated the working group on respiratory disease. During breakout sessions, participants identified key data needs in this area. They included improved measures for data quality assurance and control, validated correlations with health outcomes, and confirmation that remote-sensing data accurately represent exposures on the ground. Participants speculated that remote sensing could help fill gaps in existing exposure data and suggested a demonstration project that correlates asthma prevalence with remote-sensed measures of airborne particulates and bioallergens.

Diane Gold, an associate professor at the Harvard University School of Public Health, moderated the cardiovascular disease working group. Participants in this group identified “applications,” or health outcomes, that might be served by remote-sensing data. Among them were myocardial infarction, arrhythmia, heart failure, hypertension, and stroke, in addition to a number of subclinical outcomes such as blood pressure changes and heart rate variability. Population-level application areas were also identified; they included hospital admissions and emergency room visits. Participants concluded that resolution limits might not pose problems for chronic applications, but that acute events like myocardial infarction and stroke would be better served by higher-resolution technology.

The developmental biology working group, moderated by Beate Ritz, an associate professor of epidemiology at the University of California, Los Angeles, identified several uses of remote-sensing data to assess developmental outcomes; they included critical windows of vulnerability that occur before, during, and following parturition; acute versus chronic pollutant exposure dynamics; and the interaction of maternal and fetal genetic susceptibilities. Participants also identified data needs such as adequate temporal and spatial resolution in pollution measures, and improved identification and quantification of chemical species in air pollution.

The workshop prompted Earth and health scientists to begin a dialogue to develop web-based pilot studies that integrate existing remote-sensing data with ground-based analyses as a preliminary step toward this broader validation. The workshop generated significant enthusiasm for collaboration between NIEHS extramural researchers and scientists at the participating agencies and for the possible use of remote-sensing air quality and climate data to improve public health. Ideally, space-based measures will produce new views of air pollution and the extent of human exposure, possibly leading to better opportunities to protect public health.

## Figures and Tables

**Figure:**
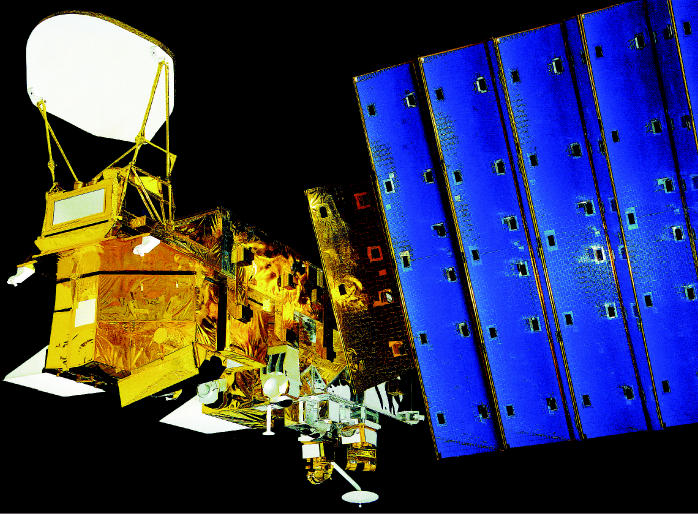
Eyes in the sky. Earth-observing satellites such as Aqua (above) are being used to monitor problems including air pollution, weather, and climate change. A recent meeting at the NIEHS brought together scientists from a broad range of disciplines to discuss how satellite data might be brought to bear on addressing issues of human health.

